# Robust inference and errors in studies of wildlife control

**DOI:** 10.1038/s41598-025-18497-7

**Published:** 2025-09-26

**Authors:** Adrian Treves, Igor Khorozyan

**Affiliations:** 1 University of Wisconsin, Madison, USA; 2 Independent Consultant, Göttingen, Germany; 3 WWF Armenia, Yerevan, Armenia; 4 Scientific Center of Zoology and Hydroecology, Yerevan, Armenia

**Keywords:** Ecology, Computational models

## Abstract

Randomized, controlled trials (RCT) are seen as the strongest basis for causal inference, but their strengths of inference and error rates relative to other study designs have never been quantified for interventions designed to prevent wildlife damage to property and game. We simulate common study designs from simple correlation to RCT with crossover design. We report rates of false positive, false negative, and over-estimation of treatment effects for five common study designs under various confounding interactions and effect sizes. We find non-randomized study designs mostly unreliable and that randomized designs with suitable safeguards against biases have much lower error rates. One implication is that virtually all studies of lethal interventions against predatory wildlife appear unreliable. Generally, applied fields can benefit from more robust designs against the common confounding effects we simulated.

**Supplementary Information:**

The online version contains supplementary material available at 10.1038/s41598-025-18497-7.

## Introduction

Identifying the cause of a phenomenon often holds the key to developing an effective intervention to interrupt the cause-and-effect connections or improve outcomes. The stakes increase whenever an intervention produces counter-productive effects on the target or side-effects for another valued entity. Therefore, scientific and public scrutiny of outcomes rather than intentions is intensifying in many applied fields^1,2^. For example, as societies attach more value to wild animals, scrutiny has intensified for interventions intended to protect human interests from wild animals. Recognition of ineffective or counter-productive effects of lethal wildlife control has exposed an alternative to the traditional hypothesis that removing wild animals, e.g., killing gray wolves (*Canis lupus*), might prevent damage to assets or resources^3^. The more recent hypothesis predicts that removing wild animals might exacerbate the losses of property or threats to safety or resources^3^. Hence, wildlife scientists have become increasingly introspective about robust study designs to evaluate the effectiveness of interventions to prevent wildlife damage to things people value, hereafter wildlife control interventions or predator control^2–5^. Resolving these uncertainties about wildlife control interventions would advance the fields of human-animal interactions and ethics, including subfields of biodiversity conservation, agricultural or other property protection, predator-prey management, and animal welfare. Other applied fields whose interventions may backfire might also benefit from such introspection.

### Quantifying the strengths of inference across study designs

Most investigators advocate the so-called ‘gold standard’ of randomized, controlled trials (RCT) without biases^6–8^. We define the following paragraphs of standards in Table S1. Yet the urgency of problems may rule against using RCT, exposing tension between swift action and well-informed action^9^. Moreover, RCT can also be opposed by interest groups^10,11^, or practically infeasible, especially for higher standard designs. When RCT are fortified by crossover (within-subject analysis including the reversal of treatment and control conditions for all subjects) and other blinding steps it may seem impractical for field practitioners to avoid research and publication biases^3^. Therefore, evaluations of the effectiveness of interventions in many fields often rely on lower standards of evidence than randomized designs^1,11,12^. Drawing inferences from studies with less robust designs than RCT is the norm in studies of wildlife or ecosystems^11,13,14^, including our field of wildlife control^3–5,14^. Approximately 75% of studies in a review of North American and European wildlife control interventions^5^, and an unquantified majority of studies in global reviews of wildlife control^14–16^ used non-randomized study designs.

Employing the convenient shorthand and ranking RCT as the gold standard, we refer to the platinum standard for crossover designs defined as above and in Table S1, and hypothesize that one could improve the strength of inference in RCT by employing a within-subjects before-and-after intervention (rBACI for randomized ‘before-after-control-impact’ design of an intervention, depending on how the authors name it^3,5,17,18^). So, we set a gold+ standard for rBACI. When non-randomized, we refer to a study design as nBACI or the ‘silver standard’. The lowest standard in this study is the ‘bronze standard’ of simple correlation, which compares different doses of intervention and outcomes.

The so-called ‘bronze standard’ lacks within-subjects comparisons so it introduces additional confounding variables of pre-existing differences between subjects (Table S1). Therefore, some authors^3,5^ predicted that the gold standard and higher would outperform the silver and bronze standards in strength of inference by a factor of two or more. They further predicted that nBACI would outperform simple correlations and rBACI would outperform RCT, but did not estimate by how much^3^.

However, randomized designs are not free of concerns^6^. Murtaugh^18^ simulated how temporal autocorrelations confounded the interpretation of a treatment effect in BACI designs employing both non-randomized and randomized designs^17^. Among the concerns, false positive rates (FPR, inferring a treatment effect when none exists) figure prominently, e.g., electric fences are routinely deemed effective in wildlife control when the evidence is fairly weak^4^. FPR are usually under-estimated due to confusion with p-values which do not tell us how often a test or intervention will fail^8,19^. Also, “new discoveries” in which the null hypothesis of no effect of an intervention is rejected, under the traditional *p* = 0.05 threshold for statistical significance, have been producing high levels of spurious findings that fail replication attempts, whether or not they use randomized study designs^1^. A short-term remedy might be to lower the threshold for significance to *p* = 0.005 for new discoveries and reserve the traditional *p* = 0.05 threshold for replication efforts^1^. But more importantly, Benjamin et al.^1^ urge all applied fields to strengthen inference through more robust study designs with safeguards against research and publication biases.

### Simulations to quantify error rates

Here we quantified error rates to compare five study designs and their strengths of inference about the effectiveness of wildlife control interventions. Specifically, we simulated predator removal, in which predators are removed so they cannot return to the study site during the study period (e.g., long-distance relocation for long periods or killing). These simulations are analogous to removal of potential predators of domestic animals but can also be generalized to any wild animals damaging anything humans value. We followed methods in^11,12^ but took them several steps further. The simulations in^12^ revealed that sample size and study design interact in a complex fashion to influence the probability of detecting true effects on population density change. Here we extended that study by holding sample size constant and investigating two sources of confounding effects. First, we investigated the influence of background interactions arising from correlations between baseline state and intervention (i.e., in our context, property loss and wildlife removal), which is analogous to self-selection or treatment bias. That is a very common interaction in predator control.

Second, we investigated the confounding effect of correlation between baseline property loss and subsequent property loss in the absence of intervention (temporal autocorrelation). This too is a common potentially confounding effect in our field because hot spots of wildlife damage have been reported in (almost) all taxa studied (reviewed in the Discussion).

We extended the results of^8,11,12^ by measuring error rates in simulations of study designs that use Pearson’s correlation coefficients when treatment effect sizes vary in magnitude and stochasticity. We used simple simulations that expose the rates of Type I errors, Type II errors and spurious correlations in which the direction of the sign of correlation is reversed when compared to the true direction of the cause and effect. We also calculated FPRs and over-estimation bias.

Our approach applies generally to many or all fields that investigate systems characterized by the baseline-intervention-outcome or state-stimulus-reaction causal relationships, including so-called natural experiments. Our simulations modeled only three parameters and their interactions:^1^ loss of asset or resource prior to intervention, analogous to the baseline/state;^2^ removal of wildlife, shortly after time t, analogous to the intervention/stimulus; and^3^ loss after intervention, analogous to the outcome/reaction.

## Methods

All variable names and definitions are presented in SM Table S1 along with definitions of study designs and models.

To test the traditional wildlife control hypothesis (negative effect of treatment) and more recent hypothesis (positive effect of treatment), we simulated losses of property such as the number of property units (e.g., domestic animals) L_t_ lost at time t, followed by the intervention as people removed W wild animals, and then we simulated losses in the next time step (L_t+1_). To simulate crossover designs, we added W at time t + 1 resulting in L_t+2_. We modeled all W and L as independent, normally distributed random, real numbers from zero to one inclusive, hereafter R. We varied background interactions (B) to mimic potential conditions in the real world (see Credibility of models below).

### Estimating Type I and II error rates

Type I errors indicate false positives (we infer an effect of treatment when none exists) and Type II errors quantify false negatives (we infer no treatment effect when one exists). We simulated separately for each type of error, and separately ran simulations again with new iterations of random numbers. We also examined extreme Type I error when the sign of correlation was reversed over the true direction of cause-and-effect. In that simulation, we also examined extreme overestimation of treatment effects by > 2SD above a positive mean treatment effect or < 2SD below a negative mean treatment effect.

In step one, we set T = 0 for no treatment effect (W x T) and assigned B = 0, -1.16, + 1.16, -2.32, or + 2.32 to vary the potentially confounding background interactions described above. We combined different background interactions for Models 0–8 to estimate rates of Type I errors (Table 1, Panels A–D). We set the coefficients empirically to yield an average Pearson’s *r* = 0.50 (*n* = 1000 replicates, 10 iterations) so there would be an equal space in either tail for errors. We simulated 200 sets of 20 correlation coefficients with *n* = 50 replicates each (400 iterations per scenario) for each of the 9 model permutations (3600 iterations per scenario-model).

In step two, we repeated the same number of independent simulations as in step one but added a treatment effect. We simulated cause-and-effect relationships between W and L_t+1_ (i.e., we set T = ± 0.58), to estimate rates of Type II errors (Table 1, Panels E–H).

For step three, we estimated FPR following^8^ as Type I error rate/[Type I error rate + (1- Type II error rate)] using data from Table 1 to construct Table 2.

**Table 1:** Error rates estimated with and without background interactions: (A) B = 1.16, (B) B = 2.32, (C, D) T = 0 for Type I error, (E–H) are set to T = 0.58 x W. Models 0–8 are described in SM Table S1.

	Simple correlation	nBACI	RCT †	rBACI †	Crossover design †	Simple correlation	nBACI	RCT †	rBACI †	Crossover design †
Models	A. Background interactions 1.16					B. Background interactions 2.32				
	C. Type I errors					D. Type I errors				
0	0.053	0.053	0.055	0.040	0.068	0.053	0.053	0.055	0.040	0.068
1	0.055	0.515				0.068	0.745			
2	0.068	0.548				0.060	0.718			
3	0.050	0.050	0.075	0.060	0.043	0.038	0.045	0.053	0.048	0.035
4	0.045	0.075	0.063	0.083	0.050	0.058	0.070	0.053	0.055	0.060
5	0.225	0.145				0.405	0.105			
6	0.223	0.595				0.435	0.743			
7	0.240	0.615				0.448	0.760			
8	0.218	0.158				0.455	0.088			
Models	E. Type II errors, positive treatment					F. Type II errors, positive treatment				
0	0.025	0.185	0.000	0.023	0.193	0.025	0.185	0.000	0.023	0.193
1	0.005	0.385				0.000	0.010			
2	0.000	0.000				0.000	0.000			
3	0.245	0.195	0.020	0.020	0.190	0.515	0.475	0.350	0.238	0.203
4	0.190	0.410	0.030	0.238	0.200	0.595	0.710	0.340	0.505	0.165
5	0.005	0.135				0.000	0.000			
6	0.210	0.915				0.365	0.890			
7	0.000	0.000				0.000	0.000			
8	0.190	0.000				0.350	0.015			
Models	G. Type II errors, negative treatment					H. Type II errors, negative treatment				
0	0.030	0.195	0.000	0.015	0.188	0.030	0.195	0.000	0.015	0.188
1	0.000	0.000				0.000	0.000			
2	0.000	0.440				0.000	0.005			
3	0.255	0.220	0.033	0.030	0.185	0.640	0.500	0.275	0.173	0.205
4	0.205	0.435	0.018	0.215	0.208	0.575	0.715	0.338	0.505	0.245
5	0.180	0.005				0.385	0.025			
6	0.005	0.005				0.005	0.005			
7	0.180	0.890				0.370	0.895			
8	0.000	0.075				0.000	0.000			

† Blank cells reflect that random assignment eliminates a correlation between W and L_t_.

**Table 2:** False positive rates (FPR) estimated from Type I and II error rates in Table 1 with background interactions: (A) B = 1.16, (B) B = 2.32, (C) positive treatment effect, (D) negative treatment effect.

	False positive rates (FPR), %									
Models	Simple correlation	nBACI	RCT †	rBACI †	Crossover design†	Simple correlation	nBACI	RCT †	rBACI †	Crossover design†
	A. Background interactions 1.16					B. Background interactions 2.32				
	C. Positive treatment ††									
0	5.2	6.1	5.2	3.9	7.8	5.2	6.1	5.2	3.9	7.8
1	5.5	45.6				6.4	42.9			
2	6.4	35.4				5.7	41.8			
3	6.2	5.8	7.1	5.8	5.0	7.3	7.9	7.5	5.9	4.2
4	5.3	11.3	6.1	9.8	5.9	12.5	19.4	7.4	10.0	6.7
5	18.4	14.4				28.8	9.5			
6	22.0	87.5				40.7	87.1			
7	19.4	38.1				30.9	43.2			
8	21.2	13.6				41.2	8.2			
	D. Negative treatment effect ††									
0	5.2	6.2	5.2	3.9	7.7	5.2	6.2	5.2	3.9	7.7
1	5.2	34.0				6.6	42.7			
2	6.4	49.5				5.7	41.9			
3	6.3	6.0	7.2	5.8	5.0	9.5	8.3	6.8	5.5	4.2
4	5.4	11.7	6.0	9.6	5.9	12.0	19.7	7.4	10.0	7.4
5	21.5	12.7				39.7	9.7			
6	18.3	37.4				30.4	42.8			
7	22.6	84.8				41.6	87.9			
8	17.9	14.6				31.3	8.1			
Minimum	5.2	5.8	5.2	3.9	5.0	5.2	6.1	5.2	3.9	4.2
95% CI of mean	9–15	17–41	5–7	4–8	5–7	13–27	18–42	6–8	5–9	5–7

† Blank cells reflect that random assignment eliminates a correlation between W and L_t_.

†† Simulated positive treatments may produce different FPR than negative treatments.

In step four, we produced five new independent simulations (400 iterations each) to investigate variations of Type I error in which the lack of a treatment effect changed from a constant T = 0 to a normally distributed random variable centered on zero but with more or less variability per subject from − 0.5 to + 0.5, -1 to + 1, -2 to + 2, -4 to + 4, and finally − 8 to + 8. This procedure simulates stochastic variability in response of subjects to the same treatment. Operationally, we created random T by subtracting two random numbers of equal magnitude from each other for every replicate. This is analogous to a treatment effect that varies by subject (see Credibility of models below).

We estimated Type I error rates again as above. We modeled with a generalized linear mixed model those error rates with four predictors (study design, variable treatment effect for each replicate, background interactions from Models 3 and 4, and the direction of Type I error - i.e., whether a spurious significant result emerged for a positive or a negative correlation).

**Fig. 1:**
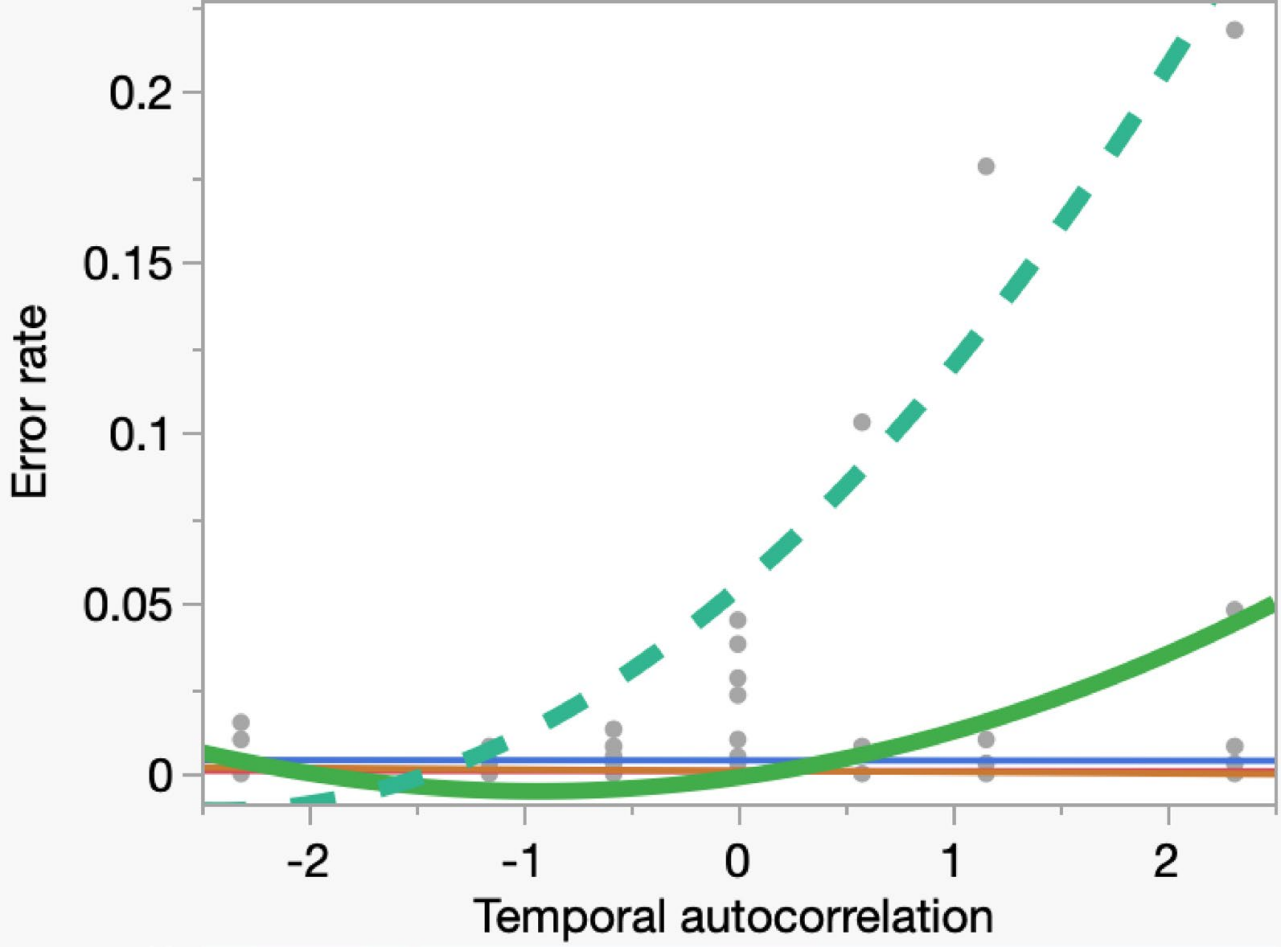
Severe Type II error resulting in reversal of the sign of correlation, in relation to temporal autocorrelation between L_t_ and L_t+1_ (B). We present a curve fit by second-order ordinary least square regression for visualization purposes only; curves for each study design (dashed green = simple correlation, solid thick green = nBACI, gold = RCT, purple = rBACI, red = crossover). The x-axis presents varying levels of temporal autocorrelation from Models 3 and 4 (Table S1 for definitions). The y-axis presents the frequency of reversal of the true sign of correlation to the opposite sign estimated from 400 iterations of each combination of study design and value of B.

**Fig. 2:**
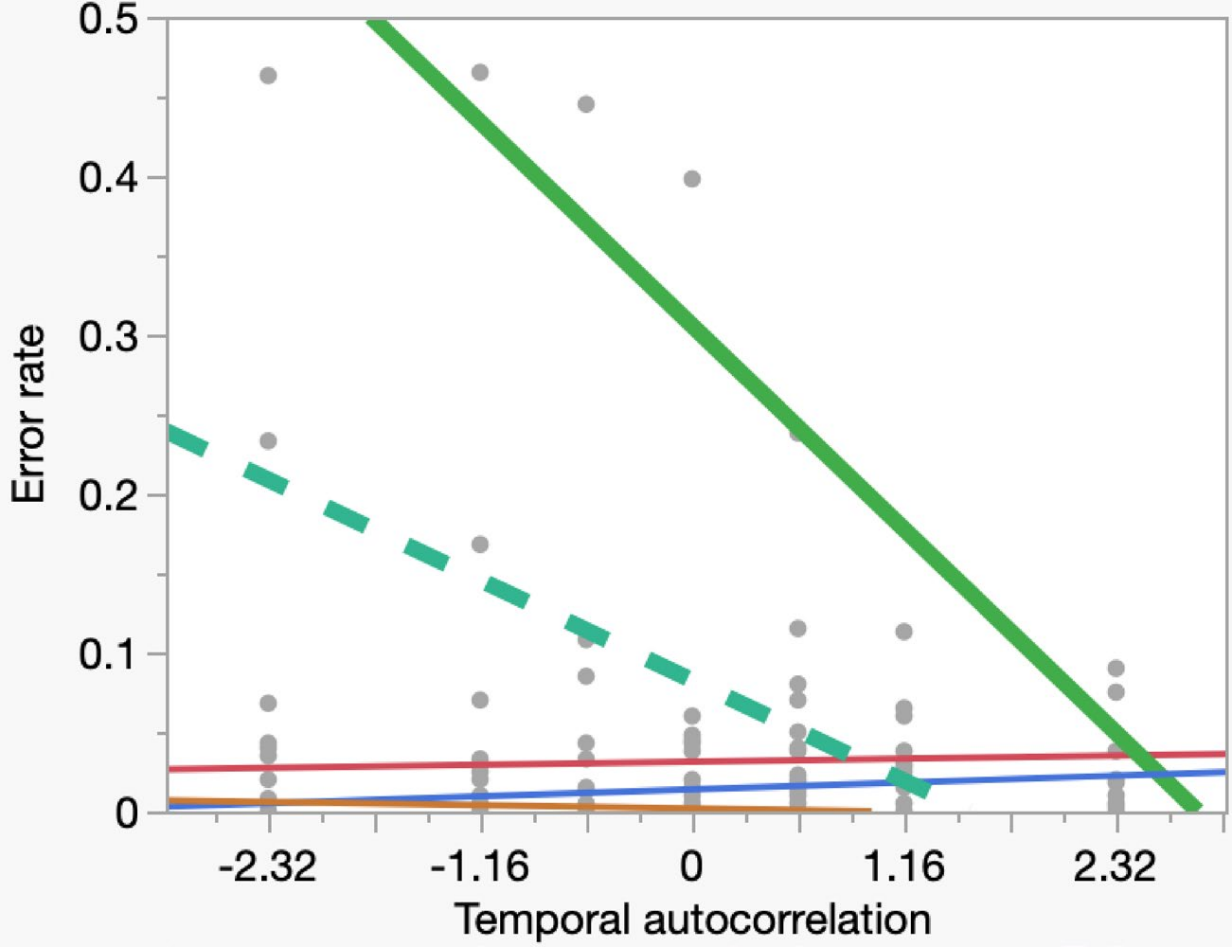
Overestimation of treatment effect in relation to temporal autocorrelation between L_t_ and L_t+1_ (B). We present a curve fit by second-order ordinary least squares regression for visualization purposes only for each study design (dashed green = simple correlation, solid thick green = nBACI, gold = RCT, purple = rBACI, red = crossover). The x-axis presents varying levels of temporal autocorrelation from Models 3 and 4 (Table S1). The y-axis presents the frequency of overestimation of treatment effect < 2SD below and > 2SD above the mean estimated from 400 iterations per data point. Simulations are the same as in Fig. 1.

In steps five and six, we explored extreme Type II errors. We ran seven simulations separate from those above (400 iterations each). For sign reversal, we counted the number of correlation coefficients that had an opposite sign as the real correlation regardless of the magnitude. In step five, for extreme errors we repeated the procedure in steps one and two but counted the number of treatment effect size estimates that exceeded the mean + 2SD for a positive treatment effect or fell below the mean − 2SD for a negative treatment effect. In step six, repeating steps three and four, temporal autocorrelation (B) varied from − 2.32 to + 2.32 independently of study design. We estimated mean and standard deviations of error rates in both steps (Figs. 1 and 2).

In all steps, we chose deterministic and probabilistic scenarios in preference to empirical rates of property loss from the literature, because the latter would include unmeasured background interactions and unreported treatment (e.g., poaching), which would undermine our effort at measuring the odds of Type I and II errors.

### Credibility of models

Background interactions simulate common situations in wildlife control. A positive correlation between W and L_t_ (Models 1 and 2, Table S1) mimics a common background interaction in which people kill more wildlife if losses were high in the past^20^. Probably uncommon, a negative correlation between W and L_t_ mimics when people kill fewer predators after high losses, e.g., when people and wildlife separate spatially after high losses^21,22^.

A positive correlation between L_t_ and L_t+1_ (Models 3 and 4, Table S1) without intervention mimics a common temporal autocorrelation, in which sites with high losses one year have high losses the next year^23,24^. Possibly less common, a negative temporal autocorrelation mimics cyclical patterns of damage in non-sequential years. For example, when wild food availability influences bear (Ursus spp.) damage to crops and human foods, one may see a negative temporal autocorrelation of losses from year to year^25,26^. Or, if predators switch from domestic to wild prey selection based on their relative scarcity or vulnerability varying over time, we can see prey switching from season to season that might produce negative autocorrelations of losses in sequential time steps^27–30^.

The above set of four background interactions create univariate permutations. In the last four bivariate permutations (Models 5–8, Table S1), we simulated both sets of interactions occurring simultaneously in a two-by-two matrix of positive or negative interactions.

For step four, when we varied the treatment effect size in every replicate, we mimicked a situation in which the same dose had variable effects on different replicates. For example, an individual predator may respond differently than its neighbor or the composition of social groups may affect how the survivors respond to removal of a group member, e.g., removing dominant individuals from a wolf pack is expected to have different effects than removing subordinate adults or pups from a pack, and even packs experiencing the same removal of dominant breeders might have different effects depending on timing and availability of replacement breeders^31,32^. Hence, the same dose (W) could have different treatment effects (T) depending on the idiosyncrasies of different replicates. Similarly, some individual predators might be attracted or repelled by vacancies left by removals of other predators^33^.

Alternately, any of the individuals involved might respond differently to lethal treatments. Theory provides five potential explanations for why the traditional hypothesis may fail^33^. In brief, the wrong predators may be killed^34^; survivors may prey on livestock that are more predictable than wild prey after the predators’ social group has been disrupted, e.g., pack hunting carnivores that rely on teamwork to hunt or reproduce successfully^35^; more immigrants may replace fewer residents that were killed^36^; smaller-bodied predator species at higher densities may refill the vacancies left by larger, scarcer predator species that died^37^; or humans and domestic animals may change their behavior after lethal intervention. When we consider the entire set of actors, wildlife, humans, and domestic animals, one can imagine inter-individual differences in response to lethal interventions. For example, some bold and tolerant individuals might explore wilder habitat after predator removal while others might continue to avoid those areas^33^. In short, the same treatment of different actors could result in diametrically opposed consequences even though the treatment did influence a subset of replicates. Despite different effects on different subjects, across replicates, the general effect of treatment approximates zero in scenarios with stochastic treatment effects. Therefore, our estimated Type I error rates illuminate FPR when treatment effects vary by subject replicate.

### Analysis

We simulated all replicates in Apple Numbers 14.3 2023. We calculated Pearson’s r in JMP Pro V17.0.0 (SAS 2023). Pearson’s r is easily interpretable, dimensionless, and suitable for normally distributed, random variables^38^. With normally distributed response variables like L and change in L, Pearson’s r is unbiased and normal (Anderson-Darling test A = 0.78, *p* = 0.05 and A = 0.37, *p* = 0.38, respectively). We calculated r in 20 batches of 50 replicates (analogous to independent sites or populations), a larger sample size than most studies of wildlife control. We used the standard critical value of |r| = 0.273 (two-tailed test at alpha = 0.05, *n* = 50 calculated from https://www.statisticssolutions.com/free-resources/directory-of-statistical-analyses/pearsons-correlation-coefficient/table-of-critical-values-pearson-correlation/, accessed 28 April 2025) in 400 iterations of each combination of scenarios (Table S1) for a total of 108,000 independent combinations. We calculated 400 correlations per simulation (108 scenarios in Tables 1 and 25 scenarios for the mixed model of Type I errors, and 35 scenarios for extreme Type II errors) for a total of 67,200 Pearson r values including 50 independent replicates each. There were fewer scenarios for randomized designs because the background interactions of L_t_ correlated with W were eliminated by random assignment procedures (Table S1).

We involved neither animals nor human subjects in this research.

## Results

### False positive rates (FPR)

Study designs differed noticeably in Type I and II error rates (Table 1) and, therefore, in FPR (Table 2). FPRs exceeded Type I error rates based on p values in 93% (100/108) of our simulations (Table 2). None of the scenarios had FPR < 1%.

The lowest FPR was 3.9% for rBACI (gold+ standard) when there were no background interactions (Table 2). In 8 scenarios, the FPR was 5.0% or less (4 scenarios with rBACI and 4 with crossover). Although rBACI had two of the lowest FPR (Table 2), it was outperformed by crossover when we introduced temporal autocorrelation in either direction, i.e., background interaction B due to correlation between L_t_ and L_t+1_. Indeed, crossover designs had a lower average FPR across 12 scenarios (6.1%, SD 1.4%) than RCT (6.4%, SD 1.0%) and rBACI (6.5%, SD 2.6%). Although these differences in FPR among randomized designs are small, the case for crossover design strengthened as we explain next.

We used a generalized linear mixed equation to model the interactions between confounding effects and study design on Type I error rates when treatment effects were centered on zero, but random in each replicate, i.e., no treatment effect in general (see Credibility of models above). The mixed model revealed significant fixed effects only for study design (df = 4, F = 78, *p* < 0.00001) and variable treatment effect for each replicate (df = 1, F = 31, *p* < 0.0001). Neither direction of error (df = 1, F = 0.2, *p* = 0.62) nor the magnitude of temporal autocorrelation (df = 6, F = 1, *p* = 0.44) were predictive of error. Also, study design and variable treatment effect for each replicate interacted significantly to predict the Type I error (df = 4, F = 64, *p* < 0.0001). Crossover performed best, because RCT and rBACI were somewhat vulnerable to randomly varying treatment effects (0.8% higher error rates), probably because the crossover design exposes each replicate to both control (treatment T = 0) and treatment (T varies randomly around zero) conditions.

By comparison to the randomized study designs, we cannot recommend simple correlation or nBACI (bronze and silver standard, respectively) because their FPR ranged from 5.2 to 42% and 5.8–88%, respectively (Table 2). Negative temporal autocorrelation (Model 4) made these designs particularly vulnerable with FPR two to three times higher than for positive temporal autocorrelation. The highest FPR arose in Models 5–8 (Table 2). Although nBACI was somewhat resistant to Models 5 and 8 when the background interactions were strong (B = 2.32), nBACI failed in most cases, including several ones with only one background interaction (Table 2). Although simple correlations yielded consistent FPR of 5-12.5% when we introduced only one background interaction, their FPR rose above 20% whenever we included two background interactions.

### Severe Type II errors: overestimation and sign reversal

Compared to randomized designs, the rates of sign reversal for simple correlation and nBACI were higher (8% and 0.8% respectively; only simple correlation differed significantly from every other design, each t-test pairwise comparison *p* < 0.0001) than randomized designs (RCT – 0.09%, rBACI – 2%, crossover – 0.08%, which did not differ among randomized designs, Welch test of unequal variances, F ratio = 2, *p* = 0.15).

Similarly, non-randomized designs had higher rates of overestimating treatment effect sizes (8% for simple correlation and 31% for nBACI), which differed significantly from randomized designs (*p* < 0.0001 for each pairwise comparison with nBACI and *p* < 0.009 for pairwise comparisons of simple correlation to each randomized design). Also, randomized study designs were statistically different in rates of overestimation error (RCT – 0.2%, rBACI – 1%, crossover – 3%, F ratio = 31, *p* < 0.0001).

As temporal autocorrelation increased, the rate of sign reversal increased and simple correlation was more strongly affected than nBACI (Fig. 1). The converse was true for overestimation error, which declined among the non-randomized study designs. Simple correlation was less prone to these errors than nBACI (Fig. 2).

**Fig. 3:**
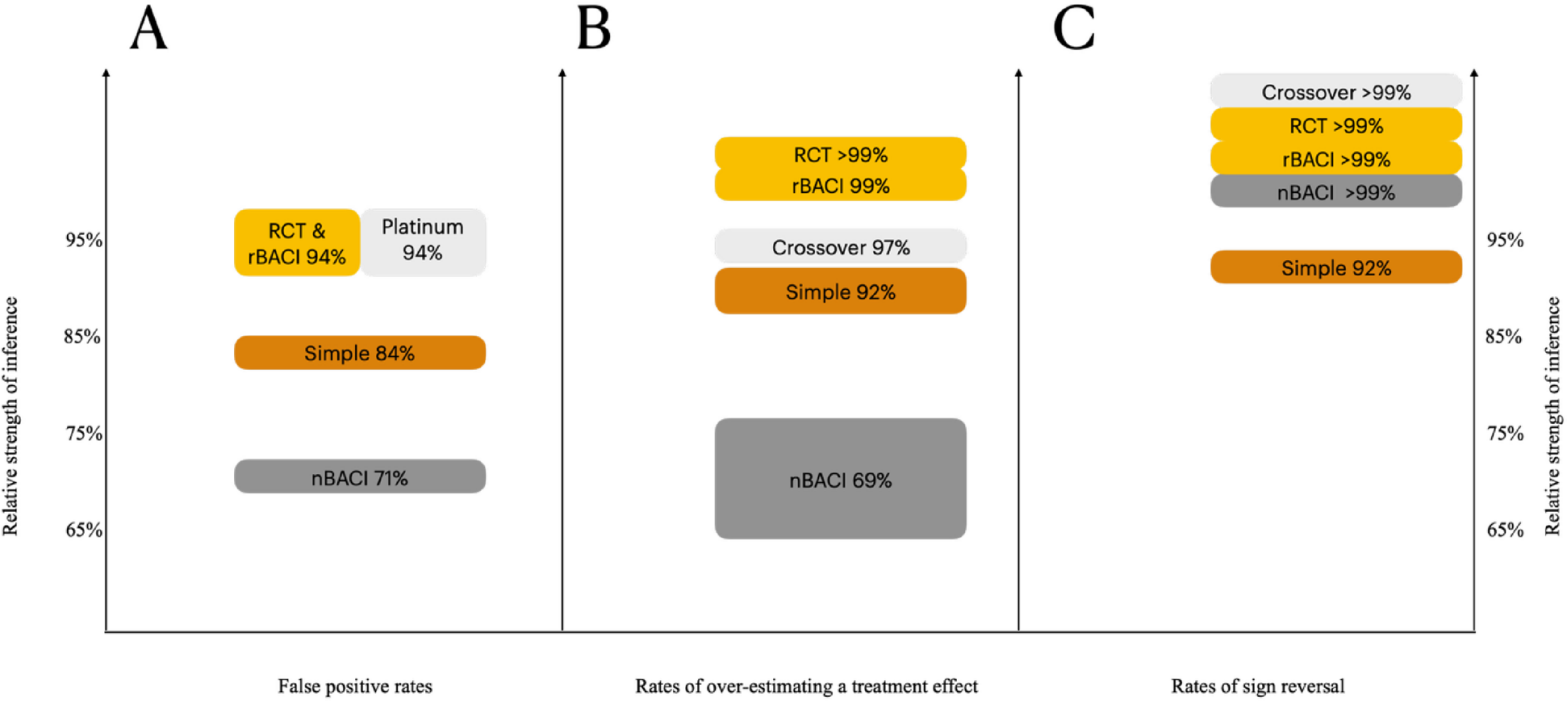
Relative strength of inference (100% - mean error rate) for crossover (platinum), RCT (gold), rBACI (gold+), nBACI (silver), and simple correlation (bronze). The height of polygons is scaled to the 95% CI within each panel: (**A**) False positive rates, (**B**) Rates of overestimating a treatment effect, and (**C**) Rates of sign reversal. Side-by-side bars (e.g., Panel A’s platinum and gold standards indicate an identical mean and 95% CI but stacked bars indicate that means were not identical (e.g., Panel C). See definitions of study designs and color codes in Table S1.

Combining results in Fig. 3, we show some predicted differences in study design and unpredicted differences.

## Discussion

We replicated published predictions that false positive rates (FPR) will generally exceed p-values. Such errors lead to spurious claims of discovery or spurious claims about the effectiveness of treatments. As predicted by^8^, our FPRs exceeded Type I error rates in > 90% of our simulations (Table 2). Therefore, we echo calls for lowering the statistical threshold for new discoveries from *p* = 0.05 down to much more conservative values, such as 0.005^1^. Regarding the strength of inference, we found support for some, but not all, of our hypotheses. As reported in^3^, study designs differed noticeably in Type I error (inferring a treatment effect when none exists) and Type II error (dismissing a treatment effect when one exists) rates (Table 1) and, therefore, in FPR (Table 2). Our results are consistent with^12^ who primarily simulated error rates in relation to sample sizes and study designs. We join them in calling for more care in randomized assignment or sampling. As with^12^, we found the randomized designs could make errors albeit far fewer than non-randomized study designs. Given FPR > 1% seems risky for strong inferences, we recommend lowering the threshold for significance level to *p* ≤ 0.01, even when randomized designs are employed.

Our results also corroborate prior cautions to measure and account for temporal autocorrelation^18^. Temporal autocorrelation is a common condition in our field because of the widespread and frequent reports of hot spots of persistent damage by wild animals year after year^23,39–42^. Some simulated Type II error rates were very high (Table 1), which by itself may not raise concern because Type II error conservatively leads to the inference of no effect when one exists. However, reporting an opposite sign of correlation than the real direction of correlation when a treatment is effective would be an extreme form of Type II error that merits concern (Fig. 1). Also, when we overestimate the real effect substantially (e.g., > 2 standard deviations above a positive mean or below a negative mean), exaggerated claims about treatment effectiveness can mislead users, payers, and distributors of that treatment (Fig. 2).

Randomized designs outperformed non-randomized designs significantly. Because Type I error rates contribute to FPR directly, the crossover design (platinum standard) provided a stronger inference than the other study designs we tested^3^. Although one might be tempted to look at a few low Type I error rates in Table 1 for simple correlation and nBACI, and declare these study designs viable in many circumstances, the FPRs in Table 2 warn against such confidence. Also, with FPR for simple correlation averaging 16% and nBACI averaging 29%, in the absence of good evidence about background interactions, one should not credit these study designs. Indeed, in many situations, particularly under field conditions surrounding wildlife control interventions, researchers will have little or no evidence to dismiss background interactions.

Here, we broaden our definition of” wildlife control” or “predator control” interventions beyond the removal of wildlife that might damage property. This is because we are aware that some of our concerns and inferences apply to interventions that do not remove wildlife but instead deter them or constrain their movements. Therefore, we use wildlife control or predator control to mean any intervention intended to prevent wildlife/predators from damaging things humans value or reduce the severity of such damage. This definition is not as broad as conflict mitigation^43,44^ or coexistence strategies^45,46^, both of which are widely discussed in our subfield. The intention to intervene (in our definition) is important to distinguish control methods from incidental features that affect vulnerability of property. By ‘things humans value’, we broaden considerations to include prey claimed by human hunters, human safety and even indirect side-effects such as stress-provoked insomnia and low livestock productivity.

Even when such evidence for background interactions is robust and well-accounted in the analyses, few researchers in our field can build a sample size of 50 on which our simulations depend. Therefore, FPR values in Table 2 are likely under-estimates of what others will encounter with smaller samples, variable treatment effects for each replicate, deviations from the assumptions of Pearson’s correlations, and measurement errors^8^.

Our predictions of the relative strength of inference among study designs were only partly supported^3^. The predicted difference between simple correlation (bronze standard) and nBACI (silver standard) held for sign reversal (Fig. 1), but not for overestimation bias (Fig. 2) or most FPR estimates (Table 2). Similarly, the so-called gold+ standard of rBACI compared to gold-standard RCT did not play out as we predicted^3^. Yet, our predictions about crossover design (platinum standard) producing stronger inference than RCT and rBACI (gold standards) were supported. Therefore, we revised our first hypotheses^3^ by producing a schematic graph of the relative strengths of inference estimated for five common study designs (Fig. 3).

Some public authorities may not test treatments with randomized, controlled trials (RCT) or similar robust experimental designs because they perceive intervening as infeasible or impractical. Perhaps decision-makers who feel accountable to the broad public, researchers and local participants might believe the treatments would be popular, but placebo controls would not^47^. Therefore, authorities may prefer to intervene in ways they consider less controversial, such as treating all subjects or serving the loudest complainants, e.g., see web panel 1 in^5^. Such steps that lead to non-randomized study designs risk backfiring with counter-productive outcomes or wasting time and resources. If fairness to human participants is a major concern, crossover designs offer participants both treatment and placebo control (randomized order) which were perceived as fair by participants in several studies of predator control^48,49^. Alternately, study participants may be assured of receiving effective interventions when the project ends^47,50,51^.

Without randomization, we see several common biases that interfere with reliable evidence and strong inference. When subjects are self-selected (self-selection bias), vulnerable subjects receive higher doses (treatment bias), or baseline conditions affect outcomes and not just treatments (e.g., temporal autocorrelation), we can expect high FPR (Fig. 3) which will result in inaccurate conclusions about interventions. Such errors are especially common for non-random before-and-after comparisons of interventions (nBACI, silver standard). Although simple correlations (bronze standard) performed better than we expected (Fig. 3), they still had unacceptably high error rates to posit predator control as reliable.

When background interactions were strong, FPR tended to rise sharply for most study designs (Table 2). When both sets of background interactions coincided, we estimated that wrong conclusions would be drawn in 18–42% of simple correlation studies and even more variably in 8–88% of non-randomized, before-and-after comparison of impacts (nBACI) studies (Table 2). Also, when temporal autocorrelation is present, the results of non-randomized study designs will produce additional errors even if the study is designed to minimize false positives. Non-randomized designs pose a considerable risk of the reversal of the sign of correlation, which can substantially mislead researchers and practitioners about the treatment effect (Fig. 1). If sign reversal does not occur, overestimation of treatment effects is also possible (Fig. 2). These compounding errors associated with non-randomized study designs can be visualized as a hierarchy of weak and strong inference (Fig. 3). We recommend no policy decision be made based on non-randomized studies of wildlife control (as we have defined it above), because drawing a conclusion that is diametrically opposed to the truth is highly likely. In such cases, inaction is preferable to hasty, unwise action.

Unlike randomized designs, non-randomized designs produce errors asymmetrical regarding positive or negative background interactions (Figs. 1 and 2). Namely, positive temporal autocorrelations produced more sign reversal errors and fewer overestimation errors in non-randomized designs than did negative temporal autocorrelations. Christie et al.^12^ also reported asymmetries (e.g., their Fig. 4b). The asymmetry we report here would tend to confuse the direction of the treatment effect more often when outcomes correlate positively to baseline conditions (Fig. 1); this situation is common in predator control where hot spots of wildlife damage recur annually (see Credibility of models in Methods). In short, whenever researchers find that losses of property recur in the same places when no interventions have occurred, they should suspect temporal autocorrelation of losses and be especially skeptical of non-randomized studies of effectiveness of interventions (Fig. 1).

Another common reason for unreliable inferences from non-randomized studies is the frequent response to losses by reactive (after the fact or ex post-facto) intervention at those properties or herds that suffered past losses. Whenever researchers note that a property suffered losses in the past and therefore received repeated intervention(s), they should suspect selection bias and be skeptical of non-randomized study designs (see Credibility of models and error rates in Table 2 when T is correlated to L_t_).

Regrettably, predator control has been dominated by non-randomized studies. Hence, predictably, there are few clear scientific consensus-based conclusions about the effects of predator control on subsequent losses, particularly in case of lethal treatments^14–16^. For example, non-randomized study designs have produced equivocal results for lethal predator control including recurrent findings of counter-productive increases in domestic animal losses following killing gray wolves^52–56^, bears^26,57,58^, and cougars (*Puma concolor*)^59,60^. Theory provides five potential explanations for why the traditional hypothesis may fail^33^.

Even well-financed RCT studies across broad areas may be hard to interpret, e.g., UK-funded RCT of badger (*Meles meles*) killing to prevent bovine tuberculosis documented variable effects of this intervention that can be difficult to detect or interpret^61–66^. Even methods considered politically unpalatable but highly effective, such as poisoning red foxes (*Vulpes vulpes*) in Australia to protect sheep, when tested with RCT prove highly variable in effect^67^. Also, there is a controversy over dingo (*Canis dingo*) killing that seems to suggest that numerous confounding variables modify desirable and undesirable effects of lethal interventions^68–70^. In Australia, the scientific consensus seems to be coalescing around a conclusion that poisoning foxes or dingoes wasted much effort and was weakly effective to ineffective because it produced very slight decreases in domestic animal mortality if the killing was not carefully regulated and monitored. Despite the above doubts, lethal methods are rarely subjected to RCT as summarized above.

More randomized studies of wildlife control in wild ecosystems have been conducted on non-lethal methods to prevent wild animals from accessing something of value to people, see these overviews^5,47,48,51,71–73^ and studies of particular species^74,75^. A small set of studies experimentally testing lethal interventions with RCTs is a concern. An analogy would be to ignore experiments on handgun control^76^, while subjecting, say, pepper spray to robust RCT. Moreover, in the absence of scientific consensus the historical intervention of killing predators continues unabated despite years of criticism^5,61^.

The resilience of lethal treatments in policy circles may reflect a perceptual bias of “cherry picking” arising from the adoption of a few effective cases and the dismissal of more numerous ineffective cases^35,55,56^. Cherry-picking examples where interventions seemed to work might arise from differential response of different subjects to similar treatments. Our mixed models show that treatments that help some replicates and harm others will raise FPR with worrying frequency in non-randomized studies. Early on, Santiago-Ávila et al.^55^ noted that perceptions of effectiveness could spread among members of the public even if the evidence for functional effectiveness is absent^77^. In addition, animal killing may fall into another perceptual bias because humans cannot recognize individual animals, some of which are culprits and some of which are not^34,35^; relatedly, some persons may claim a lethal treatment has succeeded because the death of a competitor might have been their primary goal regardless of its culpability.

If a non-randomized design is analyzed despite our cautions above, researchers should account for potential self-selection bias, treatment bias, and temporal autocorrelation. For example, lethal wildlife control studies should measure (a) killing and property losses before that killing occurred, and (b) property losses from year to year in the absence of intervention^17,18,55^. The absence of intervention includes unplanned or unregulated interventions by the people participating or using the same areas (e.g., illegal killing). This is a very difficult hurdle to overcome without strict regulation of participant actions because predator killing can still be present as an illicit behavior and hushed up^78–80^. Therefore, we suggest randomized designs in smaller, well-regulated sites are likely to be more feasible than strict experimental control over potentially confounding variables across wider regions. Even for randomized designs, we counsel care because FPR does not diminish to zero. To lower the risk of FPR, we recommend the platinum-standard crossover design RCT (all subjects receive both treatment and placebo in random order), lowering the significance threshold^1^, or other safeguards against bias^3^.

A common argument for drawing inference from non-randomized studies has been that experts can infer accurately despite confounding variables^17^. For example, expert-based adaptive managers claim they can intervene, learn, and revise without exacerbating the problems at hand and without exposing hypotheses to experimental test^2,81^. That argument depends on learning correctly. The counter-argument is that biased designs and lower standards hinder learning with false information and can produce inferences diametrically opposed to the actual effect of interventions^6,82^. Our results of sign reversal in treatment effects support the latter concern. Therefore, prioritizing randomized designs for urgent and important policy decisions may avoid the age-old problem that haste makes waste. The reasoning here provides a guide to donors, regulators, and the public to anticipate situations in which RCT becomes a prerequisite for reliable inference and sound policy.

## Supplementary Information

Below is the link to the electronic supplementary material.

Supplementary Material 1

Supplementary Material 2

## Data Availability

For scripts and a full spreadsheet with 1000 rows of data for a single iteration of each simulation, see https://faculty.nelson.wisc.edu/treves/data_archives/Simulate_study_designs_scripts_data_archive.zip, accessed August 2025.

## References

[1] Benjamin, D. et al. Redefine statistical significance. *Nat. Hum. Behav.* **2,** 6–10. https://www.nature.com/articles/s41562-017-0189-z (2018). 10.1038/s41562-017-0189-z 30980045

[2] Salafsky, N. et al. Defining and using evidence in conservation practice. *Conserv. Sci. Pract.* **1** (5), e27. 10.1111/csp2.27 (2019).

[3] Treves, A., Krofel, M., Ohrens, O. & Van Eeden, L. M. Predator control needs a standard of unbiased randomized experiments with cross-over design. *Front. Ecol. Evol.* **7**, 402–413. 10.3389/fevo.2019.00462/full (2019).

[4] Khorozyan, I. Dealing with false positive risk as an indicator of misperceived effectiveness of conservation interventions. *PLoS One* **16** (5), e0255784. 10.1371/journal.pone.0255784 (2021). 34352882 10.1371/journal.pone.0255784 PMC8342041

[5] Treves, A., Krofel, M. & McManus, J. Predator control should not be a shot in the dark. *Front. Ecol. Environ.* **14**, 380–388. 10.1002/fee.1312 (2016).

[6] Ioannidis, J. P. Why most published research findings are false. *PLoS Med.* **2** (8), e124. https://uwmadison.box.com/s/5qo4boom2r606spyxz2vsi7w835ouq7h (2005). 16060722 10.1371/journal.pmed.0020124 PMC1182327

[7] Baker, M. & Brandon, K. Is there a reproducibility crisis? A nature survey lifts the lid on how researchers view the ‘crisis’ rocking science and what they think will help. *Nature* **533**, 452–454. https://www.nature.com/articles/533452a (2016). 27225100 10.1038/533452a

[8] Colquhoun, D. An investigation of the false discovery rate and the misinterpretation of p-values. *Roy. Soc. Open. Sci.* **1**, 140216. 10.1098/rsos.140216 (2014). 10.1098/rsos.140216 PMC4448847 26064558

[9] Oreskes, N. *Why Trust Science? Princeton, NJ*. https://uwmadison.box.com/s/i2oiky9t8yqec7fww0ywdeuxduxj4m68 (Princeton University Press, 2019).

[10] Platt, J. R. Strong inference. *Science* **146**, 347–353. 10.1126/science.146.3642.347 (1964). 17739513 10.1126/science.146.3642.347

[11] Christie, A. P. et al. Quantifying and addressing the prevalence and bias of study designs in the environmental and social sciences. *Nat. Commun.* **11**, 6377. www.nature.com/naturecommunications (2020). 33311448 10.1038/s41467-020-20142-y PMC7733498

[12] Christie, A. P. et al. Simple study designs in ecology produce inaccurate estimates of biodiversity responses. *J. Appl. Ecol.* **56**, 2742–2754. 10.1111/1365-2664.13499 (2019).

[13] Clark, T. J. & Hebblewhite, M. Predator control may not increase ungulate populations in the future: A formal meta-analysis. *J. Appl. Ecol.* **58** (4), 812–824. 10.1111/1365-2664.13810 (2021).

[14] Khorozyan, I. Defining practical and robust study designs for interventions targeted at terrestrial mammalian predators. *Conserv. Biol.* **36**, e13805. 10.1111/cobi.13805 (2022). 34231934 10.1111/cobi.13805

[15] Eklund, A., López-Bao, J. V., Tourani, M., Chapron, G. & Frank, J. Limited evidence on the effectiveness of interventions to reduce livestock predation by large carnivores. *Sci. Rep.* **7**, 2097. 10.1038/s41598-017-02323-w (2017). 28522834 10.1038/s41598-017-02323-w PMC5437004

[16] van Eeden, L. M. et al. Carnivore conservation needs evidence-based livestock protection. *PLoS Biol.* **16** (9), e2005577. 10.1371/journal.pbio.2005577 (2018). 10.1371/journal.pbio.2005577 PMC6143182 30226872

[17] Underwood, A. J. & Beyond, B. A. C. I. The detection of environmental impacts on populations in the real, but variable, world. *J. Exp. Mar. Biol. Ecol.* **161**, 145–178. 10.1016/0022-0981(92)90094-Q (1992).

[18] Murtaugh, P. A. On rejection rates of paired intervention analysis. *Ecology* **83** (6), 1752–1761. 10.2307/3071993 (2002).

[19] Colquhoun, D. The reproducibility of research and the misinterpretation of p-values. *Royal Soc. Open. Sci.* **4**, 171085. 10.1098/rsos.171085 (2017). 10.1098/rsos.171085 PMC5750014 29308247

[20] Conover, M. R. Effect of hunting and trapping on wildlife damage. *Wildl. Soc. Bull.* **29** (2), 521–532. 10.2307/3784176 (2001).

[21] Knight, J. *Waiting for Wolves in Japan* (Oxford University Press, 2003).

[22] Naughton-Treves, L. Whose animals? A history of property rights to wildlife in Toro, Western Uganda. *Land Degrad. Dev.* **10**, 311–328. https://onlinelibrary.wiley.com/doi/abs/10.1002/%28SICI%291099-145X%28199907/08%2910%3A4%3C311%3A%3AAID-LDR362%3E3.0.CO%3B2-3 (1999).

[23] Treves, A., Martin, K. A., Wydeven, A. P. & Wiedenhoeft, J. E. Forecasting environmental hazards and the application of risk maps to predator attacks on livestock. *Bioscience* **61**, 451–458. 10.1525/bio.2011.61.6.7 (2011).

[24] Miller, J. R. B. Mapping attack hotspots to mitigate human–carnivore conflict: approaches and applications of Spatial predation risk modeling. *Biodivers. Conserv.* **24** (12), 2887–2911. 10.1007/s10531-015-0993-6 (2015).

[25] Garshelis, D. L. Nuisance bear activity and management in Minnesota. In: Bromley M, editor. *Bear - People Conflicts - Proceedings of a Symposium on Management Strategies. Yellowknife, Canada: Northwest Territories Department of Renewable Resources* 169 – 80. (1989).

[26] Northrup, J. M. et al. Experimental test of the efficacy of hunting for controlling human–wildlife conflict. *J. Wildl. Manage.* **87** (3), e22363. 10.1002/jwmg.22363 (2022).

[27] Janeiro-Otero, A., Newsome, T. M., Van Eeden, L. M., Ripple, W. J. & Dormann, C. F. Grey Wolf (*Canis lupus*) predation on livestock in relation to prey availability. *Biol. Conserv.* **243**10.1016/j.biocon.2020.108433 (2020).

[28] Khorozyan, I., Ghoddousi, A., Soofi, M. & Waltert, M. Big cats kill more livestock when wild prey reaches a minimum threshold. *Biol. Conserv.* **192**, 268–275. 10.1016/j.biocon.2015.09.031 (2015).

[29] Laporte, I., Muhly, T. B., Pitt, J. A., Alexander, M. & Musiani, M. Effects of wolves on elk and cattle behaviors: implications for livestock production and Wolf conservation. *PLoS One* **5** (8), e11954. 10.1371/journal.pone.0011954 (2010). 20694139 10.1371/journal.pone.0011954 PMC2915913

[30] Odden, J., Herfindal, I., Linnell, J. D. C. & Andersen, R. Vulnerability of domestic sheep to Lynx depredation in relation to roe deer density. *J. Wildl. Manage.* **72** (1), 276–282.10.2193/2005-537 (2008).

[31] Brainerd, S. M. et al. The effects of breeder loss on wolves. *J. Wildl. Manage.* **72** (1), 89–98. 10.2193/2006-305 (2008).

[32] Borg, B. L. et al. Implications of harvest on the boundaries of protected areas for large carnivore viewing opportunities. *PLoS One* **11** (4), e0153808. 10.1371/journal.pone (2016). 27124729 10.1371/journal.pone.0153808 PMC4849653

[33] Elbroch, L. & Treves, A. Why might removing carnivores maintain or increase risks for domestic animals? *Biol. Conserv.* **283**, 110106. 10.1016/j.biocon.2023.110106 (2023).

[34] Plumer, L., Talvi Tn, Männil, P. & Saarma, U. Assessing the roles of wolves and dogs in livestock predation and suggestions for mitigating human-wildlife conflict and conservation of wolves. *Conserv. Genet.* **19**, 665–672. 10.1007/s10592-017-1045-4 (2018).

[35] Knowlton, F. F., Gese, E. M. & Jaeger, M. M. Coyote depredation control: an interface between biology and management. *J. Range Manag.* **52**, 398–412. 10.2307/4003765 (1999).

[36] Cooley, H. S., Wielgus, R. B., Robinson, H. S., Koehler, G. M. & Maletzke, B. T. Does hunting regulate cougar populations? A test of the compensatory mortality hypothesis. *Ecology* **90**, 2913–2921. 10.1890/08-1805.1 (2009). 19886499 10.1890/08-1805.1

[37] Crooks, K. R. & Soulé, M. E. Mesopredator release and avifaunal extinctions in a fragmented system. *Nature* **400**, 563–566. http://www.elkhornsloughctp.org/uploads/files/1238046095Crooks_Soule_1999_Nature_Mesopredators.pdf (1999).

[38] Open Science Collaboration. Estimating the Reproducibility of Psychological Science. https://osf.io/ezcuj/2015. p. https://osf.io/ezcuj/. 10.17605/OSF.IO/EZCUJ. https://osf.io/447b3/download.

[39] Naughton-Treves, L. Predicting patterns of crop damage by wildlife around Kibale National park, Uganda. *Conserv. Biol.* **12**, 156–168. 10.1111/j.1523-1739.1998.96346.x (1998).

[40] Karanth, K. K., Gopalaswamy, A. M., Prasad, P. K. & Dasgupta, S. Patterns of human-wildlife conflicts and compensation: insights from Western Ghats protected areas. *Biol. Conserv.* **166**, 175–185. 10.1016/j.biocon.2013.06.027 (2013).

[41] Miller, J. R. B., Jhala, Y. V., Jena, J. & Schmitz, O. J. Landscape-scale accessibility of livestock to tigers: implications of Spatial grain for modeling predation risk to mitigate human–carnivore conflict. *Ecol. Evol.* **5** (6), 1354–1367. 10.1002/ece3.1440 (2015). 25859339 10.1002/ece3.1440 PMC4377277

[42] Treves, A. & Rabenhorst, M. F. Risk map for Wolf threats to livestock still predictive 5 years after construction. *PLoS One* **12** (6), e0180043. http://journals.plos.org/plosone/article?id=10.1371/journal.pone.0180043 (2017). 28665979 10.1371/journal.pone.0180043 PMC5493348

[43] Treves, A., Wallace, R. B., Naughton-Treves, L. & Morales, A. Co-managing human-wildlife conflicts: A review. *Hum. Dimensions Wildl.* **11** (6), 1–14. 10.1080/10871200600984265 (2006).

[44] Treves, A., Wallace, R. B. & White, S. Participatory planning of interventions to mitigate human-wildlife conflicts. *Conserv. Biol.* **23** (4), 1577–1587. 10.1111/j.1523-1739.2009.01242.x (2009). 19459896 10.1111/j.1523-1739.2009.01242.x

[45] Naha, D., Chaudhary, P., Sonker, G. & Sathyakumar, S. Effectiveness of non-lethal predator deterrents to reduce livestock losses to Leopard attacks within a multiple-use landscape of the Himalayan region. *PeerJ* **8**, e9544. 10.7717/peerj.9544 (2020). 32775051 10.7717/peerj.9544 PMC7384438

[46] Amit, R. & Jacobson, S. K. Participatory development of incentives to coexist with Jaguars and pumas. *Conserv. Biol.* **32** (4), 938–948. 10.1111/cobi.13082 (2018). 29356132 10.1111/cobi.13082

[47] Ohrens, O., Bonacic, C. & Treves, A. Non-lethal defense of livestock against predators: flashing lights deter Puma attacks in Chile. *Front. Ecol. Environ.* **17** (1), 32–38. 10.1002/fee.1952 (2019).

[48] Louchouarn, N. X. & Treves, A. Low-stress livestock handling protects cattle in a five-predator habitat. *PeerJ* **11**, e14788. 10.7717/peerj.14788 (2023). 36793893 10.7717/peerj.14788 PMC9924134

[49] Treves, A. et al. Gold-standard experiments to deter predators from attacking farm animals. *Anim. Front.* **14** (1), 40–52. 10.1093/af/vfad072 (2024). 38369996 10.1093/af/vfad072 PMC10873015

[50] Gehring, T. M., VerCauteren, K. C. & Cellar, A. C. Good fences make good neighbors: implementation of electric fencing for Establishing effective livestock protection dogs. *Human–Wildlife Interact.* **4**, 144–149. https://digitalcommons.usu.edu/cgi/viewcontent.cgi?article=1256&context=hwi (2010).

[51] Khorozyan, I., Siavash, G., Mobin, S., Soofi, M. & Waltert, M. Studded leather collars are very effective in protecting cattle from Leopard (Panthera pardus) attacks. *Ecol. Solutions Evid.* **1** (1), e12013. https://besjournals.onlinelibrary.wiley.com/doi/full/10.1002/2688-8319.12013#:~:text=We%20conclude%20that%20studded%20leather,other%20felids%20over%20livestock%20depredation./2688-8319.12013 (2020).

[52] Kutal, M., Duľa, M., Selivanova, A. R. & López-Bao, J. V. Testing a conservation compromise: no evidence that public Wolf hunting in Slovakia reduced livestock losses. *Conserv. Lett.* **17** (1). 10.1111/conl.12994 (2024).

[53] Šuba, J. et al. Does Wolf management in Latvia decrease livestock depredation?? An analysis of available data. *Sustainability* **15** (11). 10.3390/su15118509 (2023).

[54] Grente, O. et al. Evaluating the effects of Wolf culling on livestock predation when considering Wolf population dynamics in an individual‐based model. *Wildl. Biol.* **2024** (6), e01227. 10.1002/wlb3.01227 (2024).

[55] Santiago-Avila, F. J., Cornman, A. M. & Treves, A. Killing wolves to prevent predation on livestock May protect one farm but harm neighbors. *PLoS One* **13** (1), e0189729. https://journals.plos.org/plosone/article?id=10.1371/journal.pone.0189729 (2018). 29320512 10.1371/journal.pone.0189729 PMC5761834

[56] Krofel, M., Černe, R. & Jerina, K. Effectiveness of Wolf (Canis lupus) culling as a measure to reduce livestock depredations. *Acta Silvae Et Ligni*. **95**, 11–22. https://www.researchgate.net/publication/233792224_Effectiveness_of_wolf_Canis_lupus_culling_to_reduce_livestock_depredations (2011).

[57] Khorozyan, I. & Waltert, M. Variation and conservation implications of the effectiveness of anti-bear interventions. *Sci. Rep.* **10**, 15341. 10.1098/rsos.190826 (2020). 32948793 10.1038/s41598-020-72343-6 PMC7501236

[58] Garshelis, D. L., Noyce, K. V. & St-Louis, V. Population reduction by hunting helps control human-wildlife conflicts for a species that is a conservation success story. *PLoS One* **15** (8), e0237274. 10.1371/journal.pone.0237274 (2020). 32780755 10.1371/journal.pone.0237274 PMC7418986

[59] Laundré, J. W. & Papouchis, C. The elephant in the room: what can we learn from California regarding the use of sport hunting of pumas (Puma concolor) as a management tool? *PLoS One* **15** (2), e0224638. 10.1371/journal.pone.0224638 (2020). 32053602 10.1371/journal.pone.0224638 PMC7018503

[60] Peebles, K., Wielgus, R. B., Maletzke, B. T. & Swanson, M. E. Effects of remedial sport hunting on cougar complaints and livestock depredations. *PLoS One* **8** (11), e79713. 10.1371/journal.pone.0079713 (2013). 24260291 10.1371/journal.pone.0079713 PMC3834330

[61] Bielby, J., Vial, F., Woodroffe, R. & Donnelly, C. A. Localised Badger culling increases risk of herd breakdown on nearby, not focal, land. *PLoS One* **11** (10), e0164618. 10.1371/journal.pone.0164618 (2016). 27749934 10.1371/journal.pone.0164618 PMC5066978

[62] Donnelly, C. & Woodroffe, R. Reduce uncertainty in UK Badger culling. *Nature* **485**, 582. https://www.nature.com/articles/485582a (2012). 22660310 10.1038/485582a

[63] Donnelly, C. et al. Positive and negative effects of widespread Badger culling on tuberculosis in cattle. *Nature* **439**, 843–846. 10.1038/nature04454 (2006). 16357869 10.1038/nature04454

[64] Donnelly, C. et al. Impacts of widespread Badger culling on cattle tuberculosis: concluding analyses from a large-scale field trial. *Int. J. Infect. Dis.* **11**, 300–308. 10.1016/j.ijid.2007.04.001 (2007). 17566777 10.1016/j.ijid.2007.04.001

[65] GodfrayHCJ et al. A restatement of the natural science evidence base relevant to the control of bovine tuberculosis in great Britain. *Proc. R. Soc. B*. **280** (1768), 20131634. 10.1098/rspb.2013.1634 (2013). 10.1098/rspb.2013.1634 PMC3757986 23926157

[66] Vial, F. & Donnelly, C. Localized reactive Badger culling increases risk of bovine tuberculosis in nearby cattle herds. *Biol. Lett.* **8**, 50–53. 10.1098/rspb.2013.1634 (2012). 21752812 10.1098/rsbl.2011.0554 PMC3259956

[67] Greentree, C., Saunders, G., McLeod, L. & Hone, J. Lamb predation and Fox control in south-eastern Australia. *J. Appl. Ecol.* **37, **935 – 943. 10.1046/j.1365-2664.2000.00530.x (2000).

[68] Allen, B. L. & Hampton, J. O. Minimizing animal welfare harms associated with predation management in agro-ecosystems. *Biol. Rev. Camb. Philos. Soc.* **95** (4), 1097–1108. 10.1111/brv.12601 (2020). 32302055 10.1111/brv.12601

[69] Allen, L. R., Barnes, T. S., Fordyce, G., McCosker, K. D. & McGowan, M. R. Reproductive performance of Northern Australia beef herds. 8. Impact of rainfall and wild dog control on percentage fetal and calf loss. *Anim. Prod. Sci.* **63** (4), 388–394. 10.1071/an19430 (2020).

[70] Wallach, A. D., Ramp, D. & O’Neill, A. J. Cattle mortality on a predator-friendly station in central Australia. *J. Mammal*. **98** (1), 45–52. 10.1093/jmammal/gyw145 (2017).

[71] Shivik, J. A., Treves, A. & Callahan, M. Non-lethal techniques: primary and secondary repellents for managing predation. *Conserv. Biol.* **17** (6), 1531–1537. 10.1111/j.1523-1739.2003.00062.x (2003).

[72] Radford, C. G., McNutt, J. W., Rogers, T., Maslen, B. & Jordan, N. R. Artificial eyespots on cattle reduce predation by large carnivores. *Commun. Biology*. **3**, 430. 10.1038/s42003 (2020). 10.1038/s42003-020-01156-0 PMC7414152 32770111

[73] Nanni, A. S., del Giorgio, O., Cuadrado, L., Dip Yordanoff, A. L. & Regolin, A. L. A comprehensive framework for managing human-wildlife conflicts: the case of smallholder livestock depredation by Puma (*Puma concolor*) in the Argentine dry Chaco. *Biol. Conserv.* **310**, 111394. 10.1016/j.biocon.2025.111394 (2025).

[74] Beckmann, J. P. & Berger, J. Rapid ecological and behavioral changes in carnivores: the responses of black bear (Ursus americanus) to altered food. *J. Zool.* **261**, 207–212. 10.1017/S0952836903004126 (2003).

[75] Fersterer, P., Nolte, D., Ziegltrum, G. & Gossow, H. Effect of feeding stations on the home ranges of American black bears in Western washingto. *Ursus* **12**, 51–53. https://digitalcommons.unl.edu/cgi/viewcontent.cgi?article=1544&context=icwdm_usdanwrc (2001).

[76] Leshner, A. I. & Dzau, V. J. Good gun policy needs research. *Science* **359** (6381), 1195 (2018). 29590052 10.1126/science.aat5127

[77] Ohrens, O., Santiago-Ávila, F. J. & Treves, A. The twin challenges of preventing real and perceived threats to human interests. In: (eds Frank, B., Marchini, S. & Glikman, J.) Human-Wildlife Interactions: Turning Conflict into Coexistence. Cambridge: Cambridge University Press; 242–264 (2019).

[78] Liberg, O. et al. Shoot, shovel and shut up: cryptic poaching slows restoration of a large carnivore in Europe. *Proc. R. Soc. B* **270,** 91–98. 10.1098/rspb.2011.1275 (2012). 10.1098/rspb.2011.1275 PMC3259920 21849323

[79] Chapron, G. & Treves, A. Correction to ‘blood does not buy goodwill: allowing culling increases poaching of a large carnivore’. *Proc. R. Soc. B*. **283** (1845), 20162577. 10.1098/rspb.2016.2577 (2016). 10.1098/rspb.2016.2577 PMC5204176 28003458

[80] Santiago-Ávila, F. J. & Treves, A. Poaching of protected wolves fluctuated seasonally and with non-wolf hunting. *Sci. Rep.* **12**, e1738. 10.1038/s41598-022-05679-w (2022). 10.1038/s41598-022-05679-w PMC8810790 35110599

[81] Hone, J., Drake, V. A. & Krebs, C. J. The effort–outcomes relationship in applied ecology: evaluation and implications. *Bioscience* **67**, 845–852. 10.1093/biosci/bix091 (2017).

[82] González-González, J. et al. Trustworthiness of randomized trials in endocrinology—A systematic survey. *PLoS One* **14 **(2), e0212360. 10.1371/journal.pone.0212360 (2019). 30779814 10.1371/journal.pone.0212360 PMC6380622

