# Supplementary material for: Robust inference and errors in studies of wildlife control
Source: Sci Rep. 2025 Sep 26;15:33131. doi: 10.1038/s41598-025-18497-7 (PMC12475270; doi:10.1038/s41598-025-18497-7)
Supplement: Supplementary file 1 — Supplementary Material 1 [file 41598_2025_18497_MOESM1_ESM.docx]

**Table S1. Definitions of variables and study designs**

| **Variables** | **Definitions** |
| --- | --- |
| L_t_ | Loss of property at time t, e.g. number of killed domestic animals. We use "change in L" as a shorthand for L_t+1_ - L_t_ and so forth. |
| W | Treatment when people intervene to remove wildlife at time t (t is usually omitted except in crossover designs), e.g. number of predators killed. We mimicked a dose effect from zero to 1 in the same dimensionless units as losses. |
| R | Independent, normally distributed random numbers from zero to one. By independent, we imply we recalculated this value each time it was used. |
| T | Treatment effect multiplied by W in each replicate. Sometimes T was a constant set to ±2.32, ±1.16, ±0.58, or zero. At other times T was a normally distributed random variable centered on zero with the minimum and maximum values of ±0.5 to ±8. The numbers are the multiples of 0.58 which we empirically determined would yield a Pearson’s r correlation of 0.5, for convenience. |
| B | Background interaction strength set to ±2.32, ±1.16, ±0.58, or zero. We set background interactions (B) in eight permutations. The first four background interactions create univariate permutations (positive or negative and temporal autocorrelation between L_t_ and L_t+1_) or self-selection/treatment bias mimicked by correlation between W and L_t_. In the last four bivariate permutations, we simulated both sets of interactions occurring simultaneously in a two-by-two matrix of positive or negative interactions. We refer to the resulting Models as 0-8 are as follows: |
| **Models** | **Definitions** |
| Model 0 | No background interactions (B = 0) |
| Models 1 & 2 | L_t_ correlated to W positively and negatively respectively (self-selection bias) † |
| Models 3 & 4 | L_t_ correlated to L_t+1_ positively and negatively respectively (temporal autocorrelation) |
| Model 5 | Models 1 & 3 combined † |
| Model 6 | Models 1 & 4 combined † |
| Model 7 | Models 2 & 4 combined † |
| Model 8 | Models 2 & 3 combined † |
| **Study designs** | **Definitions** |
| Simple correlation (bronze standard) | Measures L_t+1_ after treatment W x T applied to all subjects (non-randomized) |
| Before-and-after comparison of intervention, nBACI (silver standard) | Measures change in L after treatment W x T applied to all subjects (non-randomized). †† |
| Randomized, controlled trial, RCT (gold standard) | Measures L_t+1_ after we treated approximately equal numbers of replicates with W x T as with W = 0 (experimental control). |
| Randomized BACI, rBACI (gold+ standard) | Measures change in L after we treated approximately equal numbers of replicates with W x T as with W = 0 (experimental control). †† |
| Crossover (platinum standard) | Measures change in L (L_t+2_ - L_t+1_) after random assignment followed by crossover reversal of condition for all replicates. i.e., it is a two-step rBACI so all replicates experience both conditions. ††† |
| † Models 1, 2, and 5-8 were not used for the randomized study designs because self-selection bias (a correlation between W and L t) was ruled out by random assignment to treatment and experimental control. | |
| †† Our simulations for simple correlation and nBACI can be compared directly because W, L_t_, and L_t+1_ are the same for both study designs. Likewise, RCT and rBACI use the same values of W, L_t_, and L_t+1_. When randomized treatment and experimental control are used nBACI becomes rBACI. | |
| ††† We note a shortcoming of our crossover simulation. In real crossover design experiments, there is an interval between L_t+1_ and the crossover step to restart the trial under a different condition. In that interval, L_t+1_ and W_t+1_ might change independently of treatment. Sometimes researchers intentionally include an extended wash-out period to avoid the "spillover" or contamination of one condition into a successive phase with a different condition. We assume no "spillover" after crossover. | |
