# Supplementary material for: Robust inference and errors in studies of wildlife control
Source: Sci Rep. 2025 Sep 26;15:33131. doi: 10.1038/s41598-025-18497-7 (PMC12475270; doi:10.1038/s41598-025-18497-7)
Supplement: Supplementary file 2 — Supplementary Material 2 [file 41598_2025_18497_MOESM2_ESM.zip › scripts and datasheets/SM scripts Figs1&2.docx]

Supplementary Material scripts

Scripts in Apple Numbers fotware and JMP 15.0.0 software from SAS 2019

Figure 1 script

Bivariate(

Y( :Type II 2sd or sign ),

X( :Name( "model3&4" ) ),

Fit Where( :design == "cross", Fit Line( {Line Color( {212, 73, 88} )} ) ),

Fit Where(

:design == "nBACI",

Fit Line( {Line Color( {61, 174, 70} ), Line Width( 5 )} )

),

Fit Where( :design == "rBACI", Fit Line( {Line Color( {66, 112, 221} )} ) ),

Fit Where( :design == "RCT", Fit Line( {Line Color( {204, 121, 41} )} ) ),

Fit Where(

:design == "simple",

Fit Line(

{Line Color( {40, 182, 143} ), Line Style( "Dashed" ), Line Width( 5 )}

)

),

SendToReport(

Dispatch(

{},

"1",

ScaleBox,

{Min( -2.881875 ), Max( 2.918125 ), Inc( 0.58 ), Minor Ticks( 1 )}

),

Dispatch( {}, "", AxisBox, {Select} ),

Dispatch(

{},

"Type II 2sd or sign",

TextEditBox,

{Set Text( "Over-estimate treatment effect by >2SD" )}

),

Dispatch(

{},

"Bivar Plot",

FrameBox,

{DispatchSeg( Line Seg( 6 ), {Line Width( 5 )} ),

DispatchSeg( Line Seg( 21 ), {Line Style( "Dashed" ), Line Width( 5 )} ),

Select}

),

Dispatch( {}, "", AxisBox( 2 ), {Select} ),

Dispatch(

{},

"model3&4",

TextEditBox,

{Set Text( "Temporal autocorrelation" )}

),

Dispatch(

{},

"Linear Fit design==cross",

TextEditBox,

{Set Text( "crossover" )}

),

Dispatch(

{},

"Linear Fit design==nBACI",

TextEditBox,

{Set Text( "nBACI" )}

),

Dispatch(

{},

"Linear Fit design==rBACI",

TextEditBox,

{Set Text( "rBACI" )}

),

Dispatch( {}, "Linear Fit design==RCT", TextEditBox, {Set Text( "RCT" )} ),

Dispatch(

{},

"Linear Fit design==simple",

TextEditBox,

{Set Text( "simple correlation" )}

),

Dispatch(

{},

"Linear Fit design==cross",

OutlineBox,

{Close( 1 ), Set Title( "crossover" )}

),

Dispatch(

{},

"Linear Fit design==nBACI",

OutlineBox,

{Close( 1 ), Set Title( "nBACI" )}

),

Dispatch(

{},

"Linear Fit design==rBACI",

OutlineBox,

{Close( 1 ), Set Title( "rBACI" )}

),

Dispatch(

{},

"Linear Fit design==RCT",

OutlineBox,

{Close( 1 ), Set Title( "RCT" )}

),

Dispatch(

{},

"Linear Fit design==simple",

OutlineBox,

{Close( 1 ), Set Title( "simple correlation" )}

)

)

)

Figure 2 script

Bivariate(

Y( :Type II 2sd or sign ),

X( :Name( "model3&4" ) ),

Fit Where( :design == "cross", Fit Line( {Line Color( {212, 73, 88} )} ) ),

Fit Where( :design == "rBACI", Fit Line( {Line Color( {66, 112, 221} )} ) ),

Fit Where( :design == "RCT", Fit Line( {Line Color( {204, 121, 41} )} ) ),

Fit Where(

:design == "nBACI",

Fit Polynomial( 2, {Line Color( {61, 174, 70} ), Line Width( 5 )} )

),

Fit Where(

:design == "simple",

Fit Polynomial(

2,

{Line Color( {40, 182, 143} ), Line Style( "Dashed" ), Line Width( 5 )}

)

),

SendToReport(

Dispatch( {}, "", AxisBox, {Select} ),

Dispatch(

{},

"Bivar Plot",

FrameBox,

{DispatchSeg( Line Seg( 6 ), {Line Color( {66, 112, 221} )} ),

DispatchSeg( Line Seg( 11 ), {Line Color( {204, 121, 41} )} ),

DispatchSeg(

Line Seg( 16 ),

{Line Color( {61, 174, 70} ), Line Width( 5 )}

), DispatchSeg(

Line Seg( 21 ),

{Line Style( "Dashed" ), Line Width( 5 )}

), Select}

),

Dispatch( {}, "", AxisBox( 2 ), {Select} ),

Dispatch( {}, "Linear Fit design==cross", OutlineBox, {Close( 1 )} ),

Dispatch( {}, "Linear Fit design==rBACI", OutlineBox, {Close( 1 )} ),

Dispatch( {}, "Linear Fit design==RCT", OutlineBox, {Close( 1 )} )

)

)
